# Supplementary figures and images for: In vitro activity and In vivo efficacy of Isoliquiritigenin against Staphylococcus xylosus ATCC 700404 by IGPD target
Source: PLoS One. 2019 Dec 20;14(12):e0226260. doi: 10.1371/journal.pone.0226260 (PMC6924684; doi:10.1371/journal.pone.0226260)

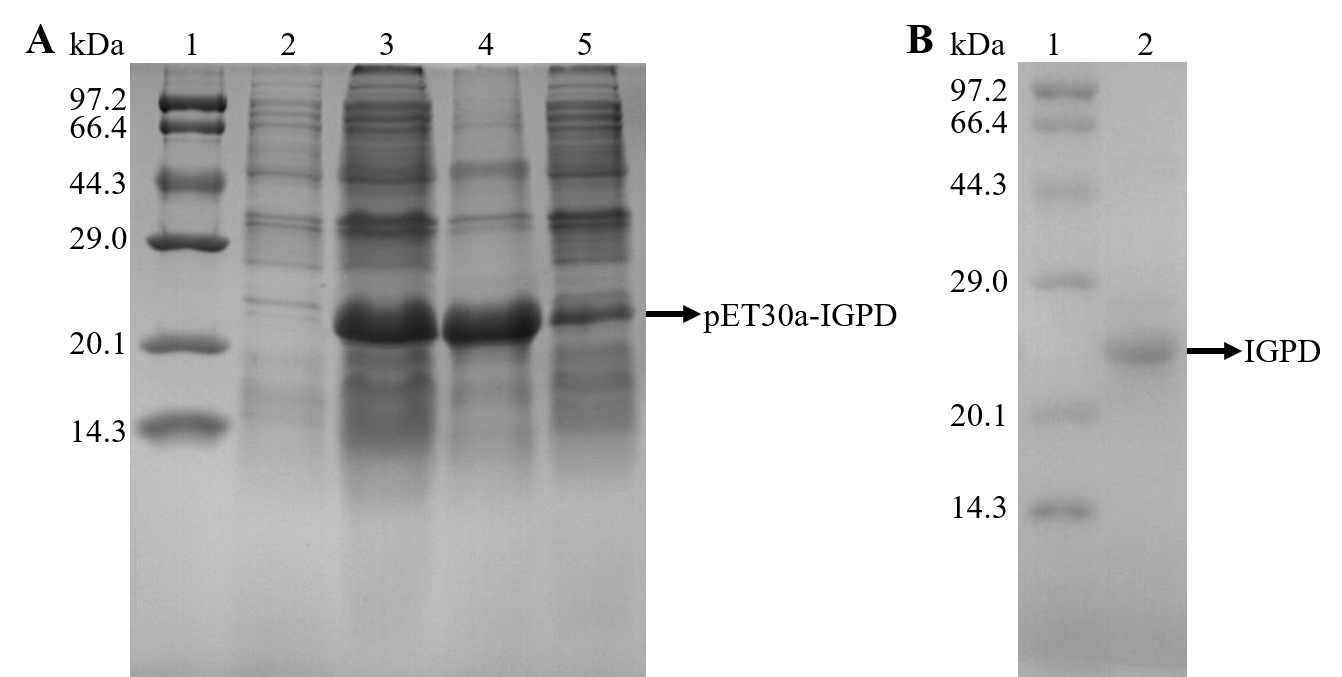

Supplement: S1 Fig — (A) SDS-PAGE gel analysis of IGPD expression as induced by the presence or absence IPTG. Lane 1: Protein molecular mass marker; lane 2: The lysates of BL21 (DE3) cells containing pET30a-IGPD without IPTG; Lane 3: The lysates of BL21 (DE3) cells containing pET30a-IGPD with IPTG; Lane 4: The supernatants of BL21 (DE3) cells containing pET30a-IGPD with IPTG; Lane 5: The precipitates of BL21 (DE3) cells containing pET30a-IGPD with IPTG. (B) SDS-PAGE gel analysis of purified IGPD. The IGPD protein was purified by a Ni Sepharose 6 Fast Flow column. Lane 1: Protein molecular mass marker; lane 2: IGPD. (TIF) [file pone.0226260.s001.tif]

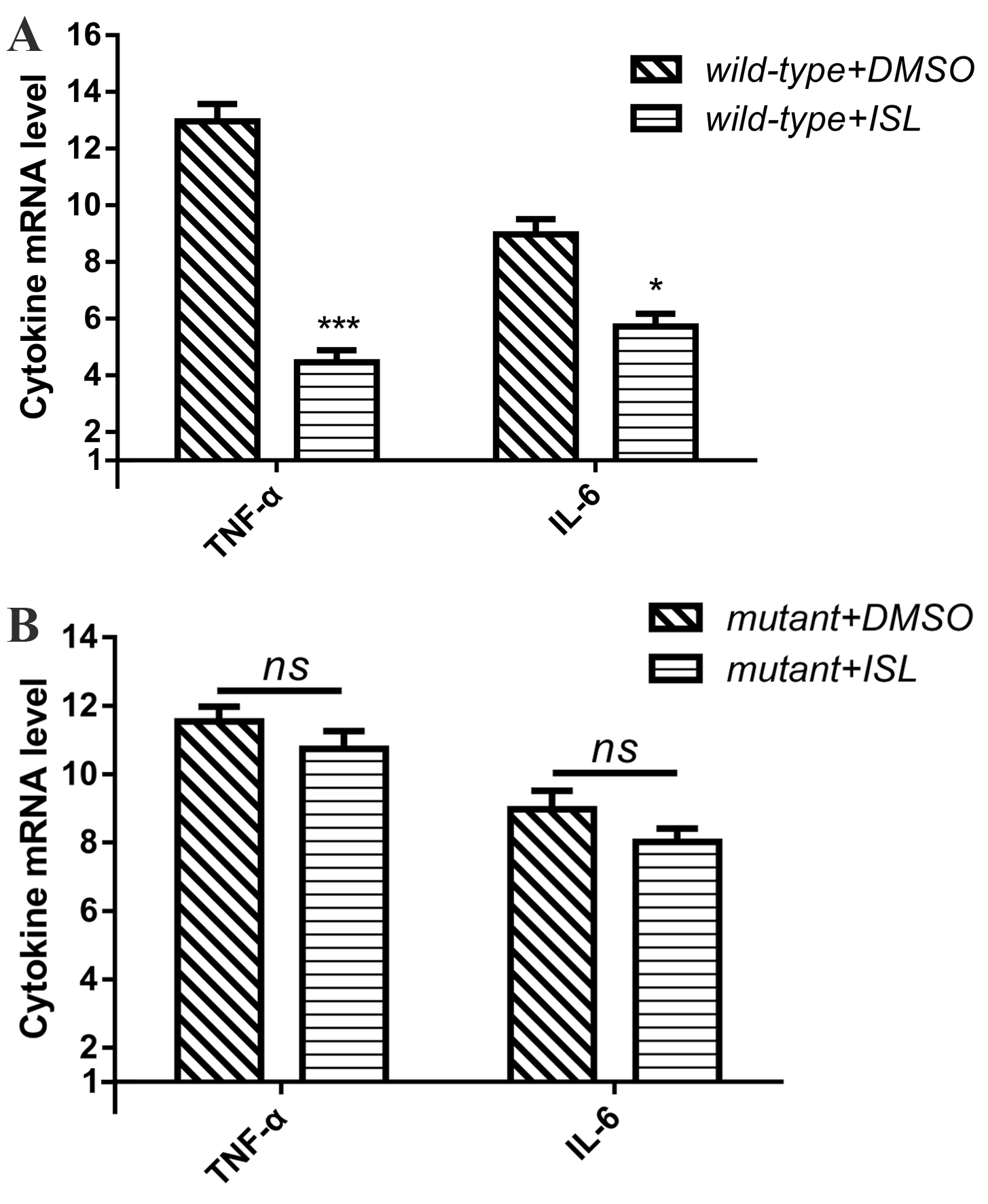

Supplement: S2 Fig — (A, B) The expression levels of cytokines, including IL-6, and TNF-α, in the mammary gland tissues of infected mice were evaluated by qPCR (n = 5) (*p < 0.05, **p < 0.01, and ***p < 0.001). (TIF) [file pone.0226260.s002.tif]
