# Supplementary material for: In vitro activity and In vivo efficacy of Isoliquiritigenin against Staphylococcus xylosus ATCC 700404 by IGPD target
Source: PLoS One. 2019 Dec 20;14(12):e0226260. doi: 10.1371/journal.pone.0226260 (PMC6924684; doi:10.1371/journal.pone.0226260)
Supplement: S1 File — (PDF) [file pone.0226260.s003.pdf]

# Original images for blots and gels

## Fig4_B_raw_images

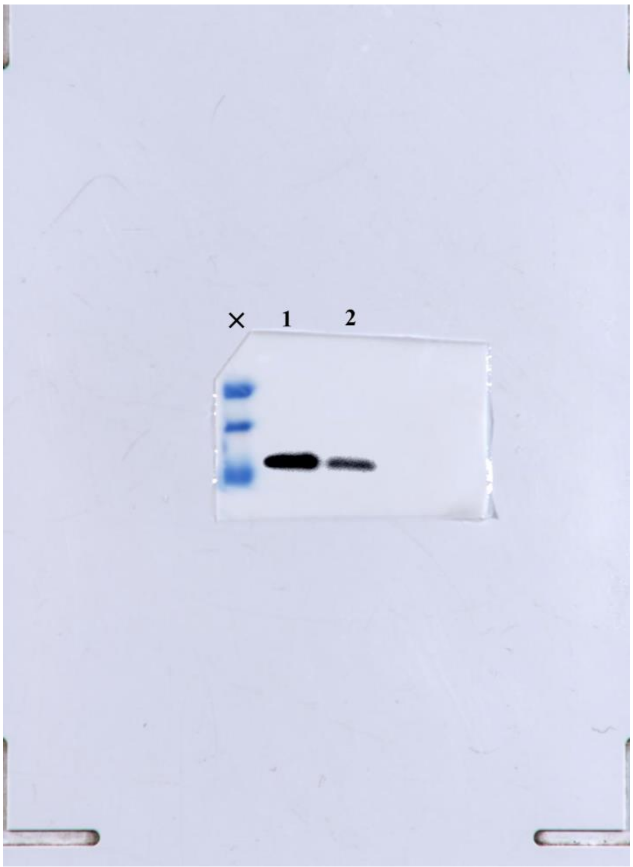


**Effect of 1/2MIC of ISL on the expression of IGPD protein in *S. xylosus*.** Line 1: Control; Line 2: 1/2MIC ISL.

# S1_A_raw_images

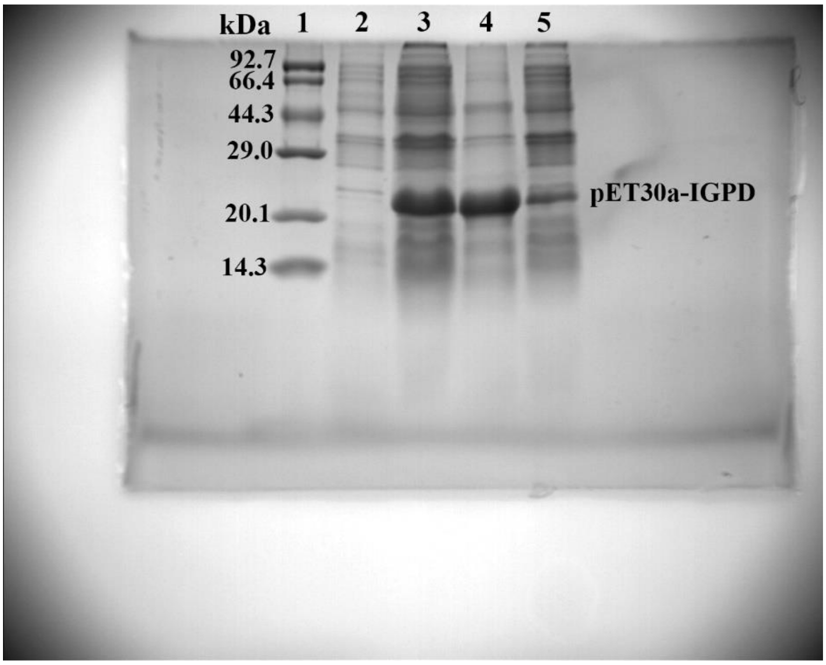


**Preparation of IGPD:** SDS-PAGE gel analysis of IGPD expression as induced by the presence or absence IPTG. Lane 1: Protein molecular mass marker; lane 2: The lysates of BL21 (DE3) cells containing pET30a-IGPD without IPTG; Lane 3: The lysates of BL21 (DE3) cells containing pET30a-IGPD with IPTG; Lane 4: The supernatants of BL21 (DE3) cells containing pET30a-IGPD with IPTG; Lane 5: The precipitates of BL21 (DE3) cells containing pET30a-IGPD with IPTG.

## S1_B_raw_images

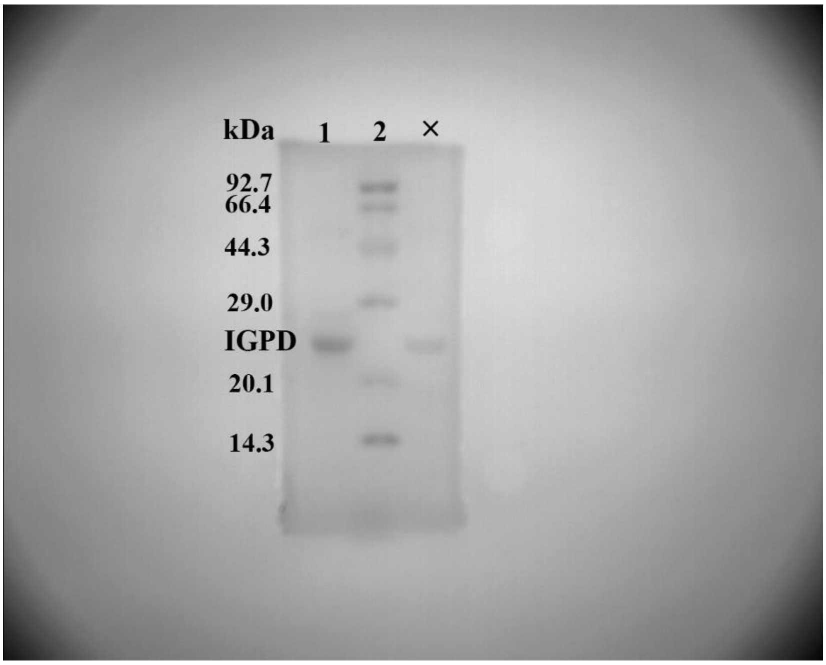


**SDS-PAGE gel analysis of purified IGPD.** The IGPD protein was purified by a Ni Sepharose 6 Fast Flow column. Lane 1: IGPD; lane 2: Protein molecular mass marker.
