# Supplementary material for: In vitro activity and In vivo efficacy of Isoliquiritigenin against Staphylococcus xylosus ATCC 700404 by IGPD target
Source: PLoS One. 2019 Dec 20;14(12):e0226260. doi: 10.1371/journal.pone.0226260 (PMC6924684; doi:10.1371/journal.pone.0226260)
Supplement: Attachment — Submitted filename: Response to Reviewers.docx [file pone.0226260.s004.docx]

**Reviewer** #1**:**

Authors of the article have prepared an excellent work, giving the full information of mode of action of using isoliquiritigenin (ISL), a flavonoid compound, as an drug in mice model mastitis. The excellence of the work is greater because authors gave a model of examination for future drugs. The subject of the work is pioneer, very important in veterinary, so I recommend it to publish.

In my opinion there is lack of a special subtitle/or more detailed describtion both tested microorganisms; I feel there was not distincly pointed what served as controls during the experiments. It would be nice if authors could slightly improve the article in methodological parts, although this feeling do not make the article worse.

1.Thank you for your suggestion and careful review. I have changed the subtitle to “Isoliquiritigenin inhibits the growth of *S.xylosus*”.

2.Thank you for your advice, I have corrected the manuscript according to your comments. I have modified the experimental method by explaining the various control groups throughout the materials and methods section of the manuscript. These corrections have been indicated in lines 100 to 101, line 109, line 207.

3.Thank you for your valuable suggestion. I have expanded and improved the materials and methods section of the article. The modified portions are indicated in line 128, line 189, lines 214 to 218.

Revised and edited portions are written in red. I hope you will be satisfied with my answer. Thank you very much.

Thank you for giving me the chance to revise my article. I have sent this manuscript to a professional English language editor to improve the language. Thank you very much for your cautious correction. Please we will welcome any other suggestions and comments that will help to further improve the quality of the paper. Thank you once again for all your and valuable contributions.

**Reviewer #2:**

Line 16 and Line 43: delete (S. xylosus)

Line 37: Italics Staphylococcus

Line 54: add ‘and’ after natural compounds

Line 120: Add reference of the studies

Line 230-233: Little more explanation of results is required

Line 296: Italicise in vitro

Line 315: delete et al

Thank you for your valuable suggestion. I have corrected some of the grammatical errors in this article. I am very sorry for my negligence.

1. I have deleted *S. xylosus* in lines 18 and 44.

2. I have italicized the *Staphylococcus* in line 37.

3. I have added ‘and’ after natural compounds in line 63.

4. I have placed the reference picture (Fig. S1) in the supplementary material in lines 127 to 128.

5. I have added some explanations of the results in lines 243 to 245.

6. I have Italicized ‘*in vitro*’ in line 308.

7. I have deleted ‘et al’ from line 325.

Revised and edited portions are written in red. I hope you will be satisfied with my answer. Thank you very much.

Thank you for the time spent in correcting my article. This has given me the opportunity to modify the work. I am grateful for all your remarks concerning the grammatical errors in the article and subsequently, the manuscript has sent to a professional English language editor for correction. I have modified the paper in line with to all your comments. Please I will be very grateful if other portions that need further improvement is brought to my notice for further correction. Thank you once again for your time.

**Reviewer #3:**

1. The authors have poorly written introduction. Likewise, in the first paragraph, the authors have given the importance of S. xylosus but in the next paragraph, they suddenly shifted to (IGPD) as the key enzymes of histidine biosynthesis and in next paragraph to flavonoids. Both these aspects are different and the authors must write in a way to easily understandable to the readers. The main aim of the study should be mentioned at the end in an elaborative manner.

2. The authors have also mentioned about the expression of IGPD in pET 30a and purification as well as preparation of its polyclonal antibody in methodology. The same should be a part of results and mentioned properly. The same results could also be a part as supplementary section.

3. The authors have reported the cytokine expression of TNF-α and IL-6. Why only two cytokines have been studied. The authors must relate the results to Th1 and Th2 type immune response based on cytokine expression. The authors must report comparative cytokine expression based on qPCR as well as ELISA as the respective results are reported based on ELISA only.

4. The entire discussion provides limited insight regarding structure-function and disease status. A more direct connection should be established by making predictions and experimental testing.

5. The authors should provide the PDB identification numbers of the structures used to generate the structural models shown and data used.

6. Throughout document (nearly every sentence) - There are numerous spelling, English grammar and syntax and typo errors. This manuscript could really benefit from English editing. For example:

7. Line 15, 34: Stapylococcus should be italics

8. Line 17: can causes

9. Line 59- main bacteria isolate

10. Line 102- meaning of ½ MIC should be mentioned

11. Line 146- Use appropriate word for “The determination was performed”

12. Line 257- it also could

13. Line 302- also can

14. Line 306-307- E. coil et al

15. Line 312- can be bind

16. Line 313- remove comma

17. Line 342-343- rewrite the sentence “There were almost no inflammatory responses or pathological changes were detected in the negative control group”

18. Line 350- remove comma

19. Line 353- has a therapeutic effective against

20. Line 22, 71, 104, 131, 297, 303 and many more - remove all sticky words

1.Thank you for your suggestion and careful review. I have expanded and improved the introduction of this article to strengthen the connection between paragraphs. This is indicated in lines 46 to 63. I have also mentioned the main purpose of the study in a relatively detailed manner in lines 72 to 77.

2.Thank you for your advice, I have corrected the manuscript according to your suggestion. I have reported the protein expression and purification results as one of the supplemental materials (Fig. S1).

3.Thank you for such a careful review.

a. I have explained the critical roles of TNF-α and IL-6 as markers of inflammation in breast tissue in lines 355 to 357.

b. In lines 366 to 371, I have linked Th1 and Th2 type immune responses to cytokine expression and conducted in-depth analysis to buttress this claim.

c. I have edited the qPCR test in lines 214 to 218, and the results are shown in the supplementary materials section (Fig. S2).

4.Thank you for your suggestion and careful review. In lines 339 to 344, I have improved my discussion of structure-function and disease status with a more detailed explanation, linking postulations with confirmatory tests.

5. I have added the PDB identification number to line 189.

6.Thank you for your valuable suggestion. I am very sorry for my negligence. I have corrected some of the syntax errors in this article.

7. I have Italicized ‘*Staphylococcus*’ in lines 17 and 37.

8., I have edited ‘can causes’ and changed it to ‘linked it with cases of’ in line 19.

9. In line 71, I have replaced ‘main bacteria isolate’ to ‘main bacterial isolates’.

10. I have replaced ‘1/2 MIC’ with ‘sub-MICs’ in line 113.

11. ‘The determination was performed’ with ‘The experiment to determine the histidine content’ in line 159.

12. I have replaced ‘it also could’ with ‘it can also ‘in line 276.

13. I have replaced ‘also can’ with ‘interfered’ in line 321.

14. I have deleted et al from line 325.

15. I have changed ‘can be bind’ to ‘can bind’ in line 330.

16. I have removed the comma from line 331.

17. I have deleted ‘There were almost no inflammatory responses or pathological changes were detected in the negative control group’ and replaced it with ‘There were no observable inflammatory responses or pathological changes in the negative control group’ in lines 363 to 365.

18. I have deleted the comma from line 376.

19. I have deleted ‘has a therapeutic effective against’ from lines 378 to 380 and replaced it with ‘This indicates that ISL has an anti-inflammatory effect by regulating the expression of TNF-α and IL-6 and reversed the damaged mammary gland and mammary epithelial cells by targeting IGPD’.

20. I have removed all sticky words including lines 24, 82, 115, 144, 316 and 322.

Revised and edited portions are written in red. I hope I have been able to correct all the issues you raised in the original manuscript satisfactorily. Thank you once again for your valuable contributions.

Thank you for giving me the opportunity to revise my article. I was particularly concerned about the final decision of this article. There may be some deficiencies in my paper, but I will be very careful to modify and improve it. I am grateful for all your remarks concerning the grammatical errors in the article. I have sent this manuscript to a professional English language editor to improve the language. I have made cautious effort to modify the paper according to all your comments. Please feel free to inform me of any portion that need further correction. Thank you once again for your time.
